# Unmet contraception need among married women in somalia: Findings from the first national health and demographic survey

**DOI:** 10.1371/journal.pone.0329491

**Published:** 2025-08-13

**Authors:** Md Badsha Alam, Md Arif Billah, Shimlin Jahan Khanam, Ibrahim Yasin Khalif, Md Nuruzzaman Khan

**Affiliations:** 1 Department of Population Science, Jatiya Kabi Kazi Nazrul Islam University, Mymensingh, Bangladesh; 2 Health Systems and Population Studies Division, Icddr, b, 68 Shaheed Tajuddin Ahmed Sarani, Mohakhali, Dhaka, Bangladesh; 3 School of Health, Medical and Applied Sciences, CQ University, Australia; 4 Faculty of Economics, Somali National University, Somalia; 5 Nossal Institute for Global Health, Melbourne School of Population and Global Health, The University of Melbourne, Australia; College of Medicine, University of Ibadan, NIGERIA

## Abstract

**Background:**

Somalia has one of the highest rates of maternal and child mortality globally, accompanied by a significantly high total fertility rate. This indicates that women in Somalia face substantial challenges in accessing family planning and contraception, although evidence on this issue remains limited. This study aims to assess the current prevalence of unmet need for contraception in Somalia and identify the socio-demographic factors associated with it.

**Methods:**

A total of 7,490 sexually active women of reproductive age with the ability to conceive were included in this study, based on pre-defined inclusion criteria, using data from the 2020 Somalia Health and Demographic Survey. The primary outcome variable was unmet need for contraception (yes/no), which included both unmet need for spacing and limiting births. A range of individual, household, and community-level variables were examined as explanatory factors. Multilevel logistic regression models were used to assess the associations between these factors and unmet need for contraception.

**Results:**

The overall prevalence of unmet need for contraception was 37.2% (95% CI: 35.3–39.1), with significant variations across socio-demographic groups. Women who married at age 21 or older (aOR: 1.25; 95% CI: 1.03–1.51), had five or more children (aOR: 2.41; 95% CI: 2.00–2.89), or lived in the central (aOR: 1.36; 95% CI: 1.05–1.76) and southern regions (aOR: 1.59; 95% CI: 1.00–2.54) were more likely to have an unmet need for contraception. In contrast, women aged 35 or older (aOR: 0.59; 95% CI: 0.43–0.79), and those residing in urban (aOR: 0.71; 95% CI: 0.52–0.98) and rural areas (aOR: 0.77; 95% CI: 0.59–0.99) were less likely to have an unmet need.

**Conclusion:**

The prevalence of unmet need for contraception in Somalia is alarmingly high by global standards. This reflects a heightened risk of short birth intervals and unintended pregnancies, contributing to increased maternal and child mortality. To address this, the government should prioritize strengthening healthcare infrastructure and implementing targeted awareness programs on family planning to reduce the unmet need for contraception in the country.

## Background

The unmet need for contraception—referring to sexually active women who wish to avoid pregnancy but are not using any contraceptive method—is a critical global issue, contributing to approximately 85 million unintended pregnancies worldwide each year [1]. This substantial number of unintended pregnancies leads to increased abortion rates and reduced utilization of maternal healthcare services, which in turn contributes to pregnancy-related complications [2,3]. The issue is particularly pronounced in countries with religious restrictions and in low- and lower-middle-income countries (LMICs), where access to safe abortion services is limited, resulting in unsafe abortion practices [4,5]. This further exacerbates the burden of unsafe abortion, adding to the already significant impact of unintended pregnancies. As a result, these combined challenges contribute to numerous adverse maternal and child health outcomes, including alarmingly high rates of maternal and child mortality [5–11]. Therefore, a high unmet need for contraception is recognized as a major barrier to achieving health-related Sustainable Development Goal (SDG) 3, particularly the targets of reducing maternal mortality to 70 deaths per 100,000 live births and child mortality to 25 deaths per 1,000 live births by 2030 [12]. Ensuring universal access to contraception and reducing the unmet need for contraception are thus essential components of the SDG framework [12].

Despite this global priority, it is alarming that 214 million women of reproductive age in LMICs still have an unmet need for modern contraception [13], representing approximately 20% of all reproductive-aged women in these countries [14,15]. The prevalence of unmet need varies across regions, with the highest levels reported in sub-Saharan Africa (23.7%) [16], followed by Latin America and the Caribbean (18%) [17]. Importantly, there are also substantial variations within countries in the same region [13]. These disparities are influenced by multiple factors including contraceptive knowledge, availability of services, and prevailing cultural and religious beliefs [18]. Socio-demographic characteristics of individuals often interact with these factors, shaping contraceptive use and access [19–21]. Thus, it is essential to explore and understand the unique contexts in which unmet need arises in order to develop effective, context-specific interventions.

Somalia, located in the Horn of Africa, has experienced decades of conflict and instability, which have had profound impacts on population health and development. With the majority of the population identifying as Muslim, Somali cultural and religious norms strongly shape attitudes toward sexual and reproductive health, particularly regarding contraception [22–26]. Many Somalis perceive contraception as contrary to Islamic teachings and believe that preventing pregnancy is sinful—a view often reinforced by religious leaders and community elders [27–29]. Additionally, low levels of education and limited access to family planning services further contribute to Somalia’s high unmet need for contraception [30–32].

Despite these concerns, there has been a lack of precise national estimates of unmet need for contraception or comprehensive analysis of its associated factors. This gap largely stems from the previous absence of nationally representative data. The dataset analyzed in this study marks the first availability of such data in Somalia, highlighting the importance of research tailored to the country’s unique cultural and socio-political context. While global and regional analyses have offered valuable insights into the determinants of unmet need [33–37], the specific cultural dynamics in Somalia require dedicated exploration. Understanding these dynamics is essential for developing targeted strategies to improve contraceptive access and utilization. Therefore, this study aims to address these gaps by examining the current prevalence of unmet need for contraception and identifying the socio-demographic factors associated with it in Somalia.

## Methods

### Data source and sampling strategy

This study utilized data from the Somali Health and Demographic Survey (SHDS) 2020, the first nationally representative survey conducted by the Government of Somalia. The survey followed the sampling methodology of the Demographic and Health Survey (DHS) program implemented by the United States [38]. A stratified multi-stage cluster sampling design was employed to collect the data. This included a three-stage sampling process in urban and rural areas and a two-stage process in nomadic areas—defined as regions inhabited temporarily by nomadic populations.

At the first stage, 55 sampling strata were initially defined across the country. Of these, 47 strata were included in the survey, while the remaining 8 strata were excluded due to security concerns. A total of 1,433 enumeration areas (EAs) were selected from the 47 eligible strata, consisting of 770 EAs from urban areas, 488 from rural areas, and 175 from nomadic areas. In the second stage, 545 EAs were selected—220 from urban strata, 150 from rural strata, and all 175 from nomadic areas. The third stage involved the selection of 30 households from each selected EA, resulting in a total of 16,360 households targeted for interviews.

**Fig 1:**
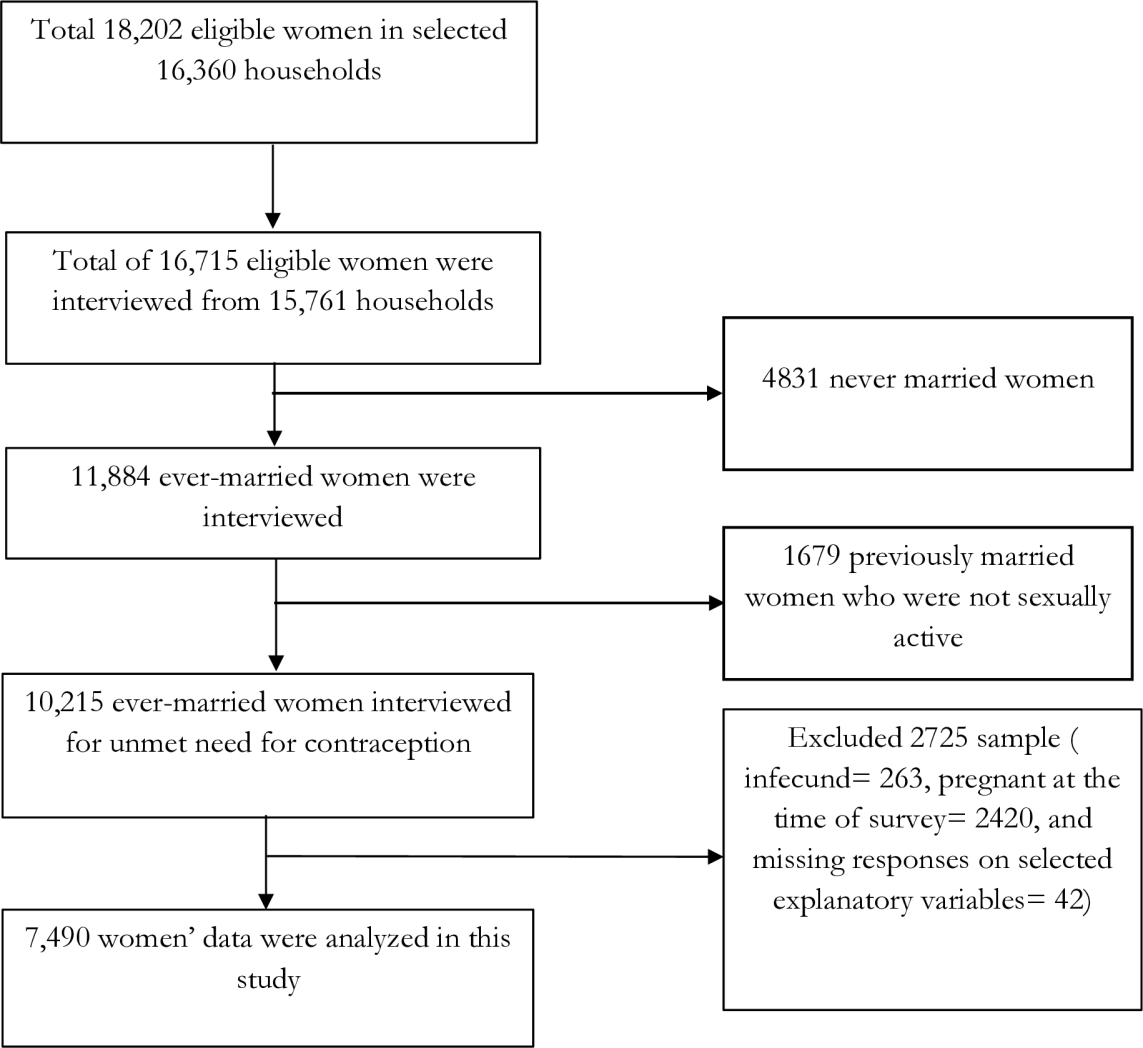
Study sample selection process to study unmet need for contraception among Somalia women, 2020.

From these households, 18,202 women were identified as eligible respondents based on the inclusion criteria: women who were either permanent or temporary residents of the selected households or who had spent the night before the survey in the household (Fig 1). Of these, 16,715 women were successfully interviewed, yielding a response rate of 91.8%. The interviews were conducted in Somali, the official language of the country, by trained enumerators using a pre-developed and pre-tested questionnaire. Further details about the survey and its sampling procedures are available in the SHDS 2020 report [38].

### Analyzed sample

A sub-sample of 7,490 women was analyzed in this study (Fig 1). These women were selected based on the following inclusion criteria: they were currently married, fecund, and sexually active; they were neither pregnant nor in the postpartum amenorrhea period at the time of the survey; and they had provided complete responses to all explanatory variables considered in the analysis.

### Outcome variable

The outcome of interest in this study was unmet need for contraception (yes or no), which includes both unmet need for spacing (women who do not wish to have a child in the near future but are not using contraception) and unmet need for limiting (women who do not want any more children but are not using contraception). Data for this variable were collected through a series of specific questions on contraceptive use and pregnancy intentions. To assess current contraceptive use, women were asked, “Are you currently using any method to delay or prevent pregnancy?” with response options coded as “yes” or “no.” Those who responded “yes” were asked a follow-up question: “What methods are you currently using to prevent pregnancy?” To assess pregnancy intentions, women were asked, “Would you like to have (a/another) child, or would you prefer not to have any (more) children?” with the response options “have (a/another) child” or “no more/none.” If a woman indicated that she wanted another child, she was then asked, “How long would you like to wait from now before the birth of (a/another) child?” Based on standard guidelines, women were classified as having an unmet need if they reported not wanting a child in the future or within the next 24 months and were not using any contraceptive method [39].

### Explanatory variables

Several socio-demographic variables were selected as explanatory factors, following a three-stage process. In the first stage, a comprehensive literature review was conducted to identify relevant factors [33–37]. In the second stage, the availability of these factors in the SHDS dataset was verified. In the third stage, the statistical significance of each available variable in relation to unmet need for contraception was assessed using chi-square tests (S1 Table, Table 1). The selected variables were categorized into individual-, household-, and community-level factors. Individual-level factors included women’s age (15–19, 20–34, and ≥35 years), age at first marriage (<18, 18–20, and ≥21 years), education (no education, primary, secondary, and higher), working status (yes or no), and parity (≤2, 3–4, and ≥5). Household-level factors included husband’s age (<24, 25–54, and ≥55 years), exposure to family planning messages via mass media (yes or no), and household wealth quintile (lowest, second, middle, fourth, and highest). The wealth quintile was derived by the survey authority using principal component analysis (PCA) of household assets, such as ownership of a radio or television, and the type of roofing material. Community-level factors included place of residence (urban, rural, and nomadic) and administrative region (Northwest, Northeast, Central, and South). The Northwest region comprised Awdal, Waqooyi Galbeed, Togdheer, Sool, and Sanaag; the Northeast region included Bari, Nugaal, and Mudug; the Central region consisted of Galgaduud, Hiraan, Middle Shabeele, and Banaadir; and the South region included Bay, Bakool, Gedo, and Lower Juba.

**Table 1:** Background characteristics and their association with unmet need for contraception, SHDS 2020 (N = 7,490).

Characteristics	Total respondents, % (95% CI)^+^	Unmet need for contraception, % (95% CI)^++^	P-value^a^
**Prevalence of unmet need for contraception**		**37.2 (35.3-39.1)**	
Unmet need for spacing		31.0 (29.5-32.7)	
Unmet need for limiting		6.2 (5.3-7.1)	
**Individual level factors**			
**Women’s age**			**<0.001**
**≤**19 years	8.0 (7.2-8.8)	29.3 (25.0-33.9)	
20-34 years	62.2 (60.4-64.0)	40.5 (38.4-42.5)	
≥35 years	29.8 (28.2-31.5)	32.5 (29.2-36.0)	
**Women’s age at first marriage**			0.427
<18 years	45.0 (42.1-47.8)	36.7 (34.1-39.2)	
18-20 years	16.0 (14.8-17.4)	35.3 (31.5-39.2)	
≥21 years	39.0 (36.3-41.8)	38.6 (35.7-41.5)	
**Women’s education**			0.157
No formal education	84.3 (82.6-86.0)	37.7 (35.7-39.7)	
Primary	11.4 (10.1-12.8)	35.6 (31.0-41.0)	
Secondary and higher	4.3 (2.4-3.5)	30.9 (25.0-39.1)	
**Women’s formal working status**			0.074
Yes	7.8 (7.0-8.7)	32.1 (27.4-37.3)	
No	92.2 (91.3-93.1)	37.6 (35.6-39.6)	
**Parity**			**<0.001**
**≤**2	30.8 (29.2-32.4)	28.3 (26.1-30.7)	
3-4	27.0 (25.6-28.5)	38.9 (35.4-42.6)	
≥5	42.2 (40.3-44.0)	40.1-44.9)	
**Husband’s age**			**0.005**
**≤**24 years	12.0 (10.7-13.4)	32.6 (28.0-37.6)	
25-54 years	77.6 (75.6-79.4)	37.9 (35.7-40.1)	
≥55 years	10.4 (9.4-11.6)	36.3 (32.3-40.5)	
**Media exposure on family planning message**			**0.030**
No	82.4 (80.8-83.9)	38.0 (36.0-40.0)	
Yes	17.6 (16.1-19.2)	33.6 (29.7-37.6)	
**Household level factors**			
**Wealth quintile**			**0.001**
Lowest	24.4 (21.3-28.0)	34.6 (31.0-38.3)	
Second	20.6 (18.5-23.0)	40.8 (37.5-44.2)	
Middle	18.0 (15.3-21.0)	40.3 (35.8-44.9)	
Fourth	19.9 (17.7-22.2)	37.8 (34.7-41.0)	
Highest	17.1 (15.0-19.5)	32.5 (28.7-36.6)	
**Community level factors**			
**Place of residence**			**0.011**
Urban	34.8 (30.7-39.1)	36.1 (33.5-38.8)	
Rural	29.8 (23.2-37.4)	36.4 (32.1-40.8)	
Nomadic	35.4 (31.1-40.0)	38.9 (36.0-41.9)	
**Administrative region**			**0.007**
Northwest	37.2 (32.1-42.6)	32.3 (29.0-35.8)	
Northeast	29.1 (23.7-35.3)	39.9 (36.8-43.1)	
Central	26.4 (22.4-30.8)	39.5 (37.1-41.9)	
South	7.3 (5.2-10.2)	42.4 (37.5-47.5)	

**Note:** All estimated percentages are weighted.

+Presented as column percentages.

++Presented as row percentages.

^a^p-values were derived from the chi-square test, which assessed the distribution of unmet need for contraception across the explanatory variables considered in the analysis. The Northwest region included Awdal, Waqooyi Galbeed, Togdheer, Sool, and Sanaag. Bari, Nugaal, and Mudug encompassed as the Northeast region. The Central region consisted of Galgaduud, Hiraan, Middle Shabeele, and Banaadir. The South region included Bay, Bakool, Gedo, and Lower Juba.

### Statistical analysis

Descriptive statistics were used to summarize the characteristics of the respondents, and Pearson chi-square tests were conducted to assess associations between unmet need for contraception and the selected explanatory variables. To identify factors associated with unmet need for contraception, multilevel modeling was employed. This approach was chosen to account for the hierarchical structure of the data, in which individuals are nested within households, and households within clusters, thereby minimizing bias in parameter estimation [38]. Four models were constructed to assess these associations. The first was a null model with no explanatory variables, serving to estimate the baseline variation in unmet need. The second model included only individual-level explanatory variables. The third model added household-level variables to the individual-level ones. The fourth and final model incorporated individual-, household-, and community-level factors. Results were reported as adjusted odds ratios (aORs) with corresponding 95% confidence intervals (CIs). All statistical tests were two-sided, and a p-value less than 0.05 was considered statistically significant. All analyses were conducted using Stata version 18.0 (StataCorp, College Station, Texas, USA).

### Ethics approval and consent to participate

The SHDS 2020 survey received ethical approval from the institutional review board of SHDS. Informed consent was obtained from all participants. All necessary patient/participant consent has been obtained, and the appropriate institutional forms have been archived. No additional ethical approval was required for this secondary analysis. Permission to access the dataset was granted, and all methods were performed in accordance with relevant guidelines and regulations.

## Results

### Background characteristics of the respondents

Table 1 presents the distribution of total respondents and the prevalence of unmet need for contraception across the explanatory variables considered. Approximately 62% of the respondents were aged between 20 and 34 years, and 45% had married before the age of 18. A substantial majority—about 85%—had no formal education, and 42% had five or more children at the time of the survey. Alarmingly, over 82% reported not receiving any family planning messages through mass media. Regarding household wealth, nearly one-quarter of the women belonged to the lowest wealth quintile. In terms of place of residence, around 35% lived in urban and nomadic areas. Regionally, the highest proportion of women resided in the Northwest (37%), followed by the Northeast (29%).

The reported prevalence of unmet need for contraception was 37.2%, with 31% attributed to unmet need for spacing and 6.2% to unmet need for limiting. The prevalence varied significantly across several explanatory variables (third column, Table 1). For example, unmet need was higher among women aged 20–34 years (40.5%), those who married at age 21 or older (38.6%), women without formal education (37.7%), and those not engaged in formal employment (37.6%).

### Factors associated with unmet need for contraception among reproductive-aged fecund women in somalia

To assess the associations between unmet need for contraception and various individual-, household-, and community-level factors, we fitted four multilevel logistic regression models (Table 2). These models were compared using Akaike Information Criterion (AIC), Bayesian Information Criterion (BIC), and intra-class correlation coefficient (ICC) values, with the best-fitting model identified as the one with the lowest AIC, BIC, and ICC values. Based on these criteria, the fourth model was selected as the optimal model (Table 2). Notably, the ICC value decreased from 24.2% in the null model to 15.4% in the final model, indicating improved model fit.

**Table 2:** Results of multilevel logistic regression models to explore factors associated with unmet need for contraception among currently married fecund women in Somali, SHDS 2020.

Characteristics	Null model aOR (95% CI)	Individual level model, aOR (95% CI)	Individual and household level model, aOR (95% CI)	Individual, household and community level model, aOR (95% CI)
**Women’s age, (ref: 15–19 years)**				
20-34 years		1.08 (0.84-1.40)	1.07 (0.83-1.37)	1.07 (0.83-1.38)
≥35 years		0.57 (0.42-0.78)^***^	0.57 (0.42-0.77)^***^	0.59 (0.43-0.79)^***^
**Women’s age at first marriage, (ref: < 18 years)**				
18-20 years		0.97 (0.79-1.20)	0.98 (0.79-1.21)	1.01 (0.84-1.23)
≥21 years		1.18 (1.00-1.40)^**^	1.19 (1.00-1.41)^**^	1.25 (1.03-1.51)^**^
**Women’s education, (ref: no formal education)**				
Primary		0.94 (0.81-1.11)	0.99 (0.84-1.16)	1.03 (0.87-1.21)
Secondary and higher		0.84 (0.59-1.20)	0.94 (0.64-1.38)	0.97 (0.65-1.44)
**Women’s working status, (ref: no)**				
Yes		0.76 (0.56-1.01)	0.76 (0.57-1.02)	0.77 (0.57-1.04)
**Parity, (ref: ≤ 2)**				
3-4		1.69 (1.42-2.00)^***^	1.67 (1.40-1.98)^***^	1.67 (1.41-1.97)^***^
≥5		2.46 (2.06-2.94)^***^	2.42 (2.00-2.94)^***^	2.41 (2.00-2.89)^***^
**Husband’s age, (ref: ≤ 24 years)**				
25-54 years			1.07 (0.80-1.44)	1.08 (0.81-1.44)
≥55 years			1.05 (0.76-1.46)	1.05 (0.76-1.45)
**Media exposure on family planning message, (ref: exposed)**				
Not exposed			1.12 (0.92-1.36)	1.08 (0.89-1.32)
**Wealth quintile, (ref: lowest)**				
Second			1.16 (0.96-1.42)	1.17 (0.94-1.45)
Middle			1.22 (0.93-1.62)	1.35 (0.87-2.10)
Fourth			1.10 (0.87-1.40)	1.25 (0.84-1.86)
Highest			0.93 (0.74-1.16)	1.08 (0.77-1.53)
**Place of residence, (ref: nomadic)**				
Urban				0.71 (0.52-0.98)^**^
Rural				0.77 (0.590.99)^**^
**Region, (ref: Northwest)**				
Northeast				1.28 (1.00-1.63)
Central				1.36 (1.05-1.76)^**^
South				1.59 (1.00-2.54)^**^
**Model summary**				
Cluster-level variance (SE)	0.98 (0.23)	0.82 (0.21)	0.68 (0.19)	0.52 (0.18)
Intra-class correlation coefficient (ICC)	24.2%	18.2%	16.3%	15.4%
Proportional change in variance (PCV)	**Reference**	24.8%	32.6%	36.3%
AIC	7556.23	6882.36	5884.21	4887.62
BIC	7558.90	6489.25	54496.23	4872.36
Constant	0.58 (0.55-0.62)	0.31 (0.22-0.45)	0.29 (0.19-0.45)	0.27 (0.18-0.40)

**Notes:**

***p < 0.01,

**p < 0.05.

^a^OR: Adjusted Odds Ratio. Ref: Reference Categories. CI: Confidence Intervals. AIC: Akaike’s Information Criterion. BIC: Bayesian Information Criterion. The Northwest region included Awdal, Waqooyi Galbeed, Togdheer, Sool, and Sanaag. Bari, Nugaal, and Mudug encompassed as the Northeast region. The Central region consisted of Galgaduud, Hiraan, Middle Shabeele, and Banaadir. The South region included Bay, Bakool, Gedo, and Lower Juba.

Several factors were significantly associated with unmet need for contraception. Women aged ≥35 years were 41% less likely to experience unmet need compared to those aged ≤19 years (aOR: 0.59; 95% CI: 0.43–0.79). Women who married at age ≥ 21 had 1.25 times higher odds of unmet need compared to those married before age 18 (aOR: 1.25; 95% CI: 1.03–1.51). The likelihood of unmet need increased with parity: women with 3–4 children had 1.67 times higher odds (aOR: 1.67; 95% CI: 1.41–1.97), while those with ≥5 children had 2.41 times higher odds (aOR: 2.41; 95% CI: 2.00–2.89) compared to women with two or fewer children.

Compared to women in nomadic areas, the likelihood of unmet need was 29% lower among urban residents (aOR: 0.71; 95% CI: 0.52–0.98) and 23% lower among rural residents (aOR: 0.77; 95% CI: 0.59–0.99). Regionally, women living in the Central region had significantly higher odds of unmet need (aOR: 1.36; 95% CI: 1.05–1.76), as did those in the South region (aOR: 1.59; 95% CI: 1.00–2.54), compared to women in the Northwest region.

## Discussion

The objective of this study was to investigate the prevalence of unmet need for contraception in Somalia and identify associated factors. The study revealed an overall prevalence of unmet need for contraception at 37.2%. This prevalence varied across different explanatory variables, with the highest level reported among women not engaged in any formal employment compared to those engaged in formal employment (37.6% vs. 32.1%). Furthermore, the odds of unmet need for contraception were higher among women who married at ≥21 years, women with parity greater than two, and women residing in the Central and South regions. Conversely, women aged ≥35 years and those residing in rural and urban areas reported lower odds of unmet need for contraception. These findings highlight the importance of addressing unmet need for contraception in Somalia. They also suggest that the implementation of the Millennium Development Goals (MDGs) between 2000 and 2015, which emphasized reducing unmet need, did not significantly contribute to increasing contraceptive uptake. This presents a serious risk to Somalia’s ability to achieve the SDG), particularly the targets related to universal access to sexual and reproductive healthcare services and reductions in maternal and child mortality. In response to these risks, the Federal Ministry of Health and the Ministry of Health and Human Services have been prioritizing healthcare service needs, including maternal health and family planning [40,41]. However, progress has been minimal. These findings underscore the urgent need for targeted interventions and policies by the government to address the high prevalence of unmet need for contraception in Somalia.

The findings of this study position Somalia among countries with the highest levels of unmet need for contraception globally. Comparable rates have been observed in countries such as Rwanda and Kenya (14%) [42,43], Benin (38%) [44], Ghana (35.17%) [45], Papua New Guinea (32.2%) [46], Somaliland (30.4%) [47], Ethiopia (34.9%) [48], Angola (42.6%) [49], as well as São Tomé and Príncipe (38%) [50], and Burundi (32.4%) [51]. A recent study in Somalia using the same survey data we analyzed, as well as the survey itself, revealed that only 7% of the population is using contraception, and among them, less than 1% reported using modern contraceptive methods [52]. The high level of unmet need, despite very low contraceptive use, and evidence that many Somali women consider a family size of six or more to be ideal (with a current total fertility rate [TFR] of 6.9), suggest that the issue stems more from limited access to contraception rather than lack of demand. This limited access can be attributed to several challenges, including restricted access to mass media, widespread community and religious misconceptions about contraception, a shortage of healthcare facilities and adequately trained providers, as well as misunderstandings about fertility, birth spacing, and family planning [38]. According to the World Health Organization, Somalia’s progress toward achieving the MDG and SDG targets related to reproductive health has been limited due to prolonged conflict, weak health infrastructure, and socio-political instability. However, localized successes have been observed in urban areas, where various reproductive health programs supported by NGOs are in place [53].

The study findings indicate a higher likelihood of unmet need for contraception among women who marry at a later age, have higher parity, and reside in the Central region. These patterns are similar to those observed in many LMICs and African nations [46,51]. Therefore, addressing these socio-demographic factors and geographical disparities is essential for promoting increased uptake of contraception. In LMICs, unmet need among women who marry later often correlates with higher levels of education. This education enhances their awareness of, and the significance of, contraception for maintaining a healthy pregnancy and delivery [50]. However, in Somalia, as revealed in this study, women’s education does not appear to impact unmet need for contraception. This is likely because female literacy stands at just 22%, and sex education is hardly featured in school curricula—resulting in no meaningful role of education in influencing contraception uptake [54]. In response to these challenges, development partners—including UNFPA—are currently working with the Somali government and other stakeholders to integrate family planning, contraception, and pregnancy-related topics into the education system [55]. This finding underscores the necessity of integrating family planning and contraception education into mainstream educational programs.

Conversely, women with three or more children expressed a strong desire for contraception to prevent further pregnancies [36]. This finding aligns with the high TFR in Somalia and suggests that lack of contraception contributes significantly to elevated fertility levels. This issue may be attributed to inadequate performance by healthcare facilities in meeting the demand for contraception or limited access due to cultural or religious barriers [38,52]. This group of women likely represents the younger generation, which shows increasing levels of education and awareness regarding contraception [38]. The study also found that older individuals were less likely to express a need for contraception, indicating a slow transition away from intergenerational, hereditary family norms [51]. Furthermore, the study examined variations in unmet need across different places of residence and regions. These disparities may result from unequal family planning service coverage or uneven socio-economic development [38]. Previous studies have shown dramatic differences in family planning coverage between regions, and between urban, rural and nomadic areas in Somalia [38,52]. Thus, advancing family planning services requires a tailored, region-specific approach. Taken together, these findings emphasize the importance of enhancing women’s education and ensuring equitable access to contraception, as both have the potential to improve uptake of family planning services in Somalia.

At a broader level, these findings can be attributed to three key factors: (i) limited access to contraception, (ii) cultural barriers, and (iii) insufficient knowledge about contraception [24,26–29,52]. These factors collectively highlight the need for comprehensive policies and programs to reduce unmet need for contraception in Somalia. A primary focus should be placed on strengthening healthcare facilities, which play a crucial role in providing access to contraception at the community level, promoting the importance of contraceptive use, and addressing cultural and religious barriers that hinder utilization [5,8]. However, addressing these issues is especially challenging for the Somali government, given that its healthcare system is among the weakest in the world, with only 34% of the population having access to essential healthcare services [38,56]. The main constraint is inadequate government funding for health, which cannot be fully addressed by international donors alone [38]. The situation is even more critical for family planning services, which require continuous community-level engagement to be effective [52]. These services are nearly impossible to deliver with the current healthcare infrastructure in Somalia due to shortages of both manpower and equipment, which are already inadequate to meet basic healthcare needs [38].

Addressing these issues will require the Somali government to increase healthcare funding and implement educational policies to train and expand the healthcare workforce [52]. However, such reforms are long-term processes and cannot be achieved overnight. In the interim, Somalia could consider adapting globally recognized models that have proven effective elsewhere, with modifications suited to the local context. For example, Bangladesh has implemented a model in which a separate Directorate under the Ministry of Health ensures the delivery of family planning and contraceptive services [57]. Secondary-educated women were recruited under this directorate, trained, and tasked with visiting every couple’s home within a small geographical area every 14 days [57]. Ethiopia has also improved reproductive health through the use of mobile clinics, which Somalia could consider adopting [58,59]. These strategies align with previous findings indicating that Somali women prefer home-based counselling and female health workers for receiving family planning information [60]. By adopting similar approaches, the Somali government could address family planning issues more effectively at the grassroots level, using generally educated women, and allowing expert healthcare personnel to focus on providing other essential health services.

This study possesses several strengths as well as a few limitations. First, it is important to note that this is the first nationally representative study conducted in Somalia, offering valuable insights into current estimates of unmet need for contraception and its determining factors. The study used internationally accepted guidelines to classify the outcome variable and applied rigorous data analysis techniques, including advanced statistical methods, while controlling for a wide range of confounders. Consequently, the findings are robust and can guide the Somali government and its development partners in formulating evidence-based policies and programs to improve contraceptive uptake. However, the study is limited by its use of cross-sectional data, which restricts the ability to infer causal relationships. Additionally, as the data were collected retrospectively, there is a possibility of recall bias. Social and religious norms may also have caused underreporting of contraceptive use. Moreover, crucial factors related to health facility availability and religious influence could not be included in the analysis due to the absence of relevant data in the survey. Despite these limitations, the study offers the first nationally representative evidence on this topic in Somalia and can inform the design of effective, targeted interventions.

## Conclusion

The prevalence of unmet need for contraception in Somalia is notably high, standing at 37.2%, placing the country among those with the highest rates globally. Moreover, the rate varies across several socio-demographic factors examined in the analysis. The study identified a greater likelihood of unmet need among women who married at age ≥ 21, those with more than two children, and women residing in the Central region. In contrast, women aged ≥35 years and those living in rural and urban areas were less likely to report unmet need for contraception. To address this issue, it is essential to strengthen the healthcare system while simultaneously promoting initiatives such as increasing educational enrollment and implementing awareness-raising programs on the importance of contraceptive use. Somalia can take inspiration from the successful family planning model implemented in Bangladesh, where generally educated women were trained and empowered to provide contraceptive services. This community-based approach enabled healthcare professionals to focus on delivering other essential services. By adopting similar strategies, Somalia can make substantial progress in reducing the prevalence of unmet need for contraception and improving overall reproductive health outcomes.

## Supporting information

S1 Table Operational definitions of the outcome and explanatory variables. (DOCX)

## Abbreviations

SHDS: Somali Health and Demographic Survey
PSUs: Primary Sampling Units
LMICs: Low- and Middle-Income Countries
