# Supplementary material for: Unmet contraception need among married women in somalia: Findings from the first national health and demographic survey
Source: PLoS One. 2025 Aug 13;20(8):e0329491. doi: 10.1371/journal.pone.0329491 (PMC12349696; doi:10.1371/journal.pone.0329491)
Supplement: S1 Table — (DOCX) [file pone.0329491.s001.docx]

**Table 1: Operational definitions of the outcome and explanatory variables.**

| Variables | Operational Definition |
| --- | --- |
| Unmet need for contraception | Refers to fecund women who are not using any contraceptive method but wish to either space or limit future pregnancies. This variable was categorized as yes or no. |
| Individual level factors |  |
| Women’s age | Age of the woman at the time of the survey, categorized as: 15–19, 20–34, and ≥35 years. |
| Women’s age at first marriage | Age at first marriage, categorized as: <18 years, 18–20 years, and ≥21 years. |
| Women’s education | Educational attainment, categorized as: no education, primary education, and secondary or higher education. |
| Women’s working status | Participation in income-generating activities, classified as: yes or no. |
| Parity | Number of children ever born to the respondent at the time of the survey, categorized as: ≤2, 3–4, and ≥5 children. |
| Household level factors |  |
| Husband’s age | Age of the respondent’s husband at the time of the survey, categorized as: <24 years, 24–54 years, and ≥55 years. |
| Media exposure to family planning messages | Indicates whether the woman had heard or seen a family planning message through media (television, radio, or newspaper/magazine) in the past few months. Categorized as: exposed or non-exposed. |
| Wealth quintile | A composite index developed by the SHDS based on household assets and characteristics. Categorized as: lowest, second, middle, fourth, and highest. |
| Community level factors |  |
| Place of residence | Place of residence at the time of the survey, classified as: urban, rural, or nomadic. |
| Administrative region | Geographic region of residence at the time of the survey, categorized as: - Northwest: Awdal, Waqooyi Galbeed, Togdheer, Sool, Sanaag - Northeast: Bari, Nugaal, Mudug - Central: Galgaduud, Hiraan, Middle Shabeele, Banaadir - South: Bay, Bakool, Gedo, Lower Juba |
